# State-dependent control of the respiratory pattern and coupled oscillators

**DOI:** 10.1186/1471-2202-12-S1-P308

**Published:** 2011-07-18

**Authors:** Ilya A Rybak, Ana PL Abdala, Yaroslav I Molkov, Julian FR Paton, Jeffrey C Smith

**Affiliations:** 1 Department of Neurobiology & Anatomy, Drexel University College of Medicine, Philadelphia, PA 19129, USA; 2 Department of Physiology & Pharmacology, School of Medical Sciences, University of Bristol, Bristol BS8 1TD, UK; 3 National Institute of Neurological Disorders and Stroke, National Institutes of Health, Bethesda, MD 20892, USA

## 

The respiratory rhythm and motor pattern are generated by a spatially organized brain stem respiratory network with a rhythmogenic core comprising interacting neural populations within the pre-Bötzinger (pre-BötC) and Bötzinger (BötC) complexes controlled by drives and inputs from other brain stem compartments. Our previous large-scale computational model [3,4] reproduced the behavior of this network under multiple natural and experimental conditions, but did not consider neural oscillations that were proposed to emerge within the retrotrapezoid nucleus / parafacial respiratory group (RTN/pFRG) and drive pre-inspiratory (or late-expiratory, late-E) oscillations in the abdominal motor output [2]. These late-E oscillations usually emerge with increasing metabolic demands (e.g., during hypercapnia or hypoxia) and couple with the respiratory oscillations generated by the BötC-pre-BötC core of the respiratory central pattern generator [1,2].

In our experiments, performed in the arterially perfused *in situ* rat preparation, the respiratory motor activity was recorded simultaneously from abdominal, phrenic, hypoglossal and central vagus nerves. Under normal conditions (eupnea), the abdominal motor outflow showed only a low-amplitude post-inspiratory (post-I) activity. An expression of the abdominal late-E bursts, preceding and coupled to phrenic discharges, occurred during hypercapnia (7–10% CO2) and could be abolished by pharmacological inactivation of the RTN/pFRG region and by administration of riluzole, a persistent sodium current (*I*_NaP_) blocker, hence confirming that the late-E oscillations originate in RTN/pFRG and involve intrinsic, *I*_NaP_-dependent mechanisms. Using these data we extended our computational model by incorporating in the RTN/pFRG compartment an additional late-E population (with neurons containing *I*_NaP_) serving as a source of abdominal late-E activity. The proposed interactions between the BötC’s and pre-BötC’s populations and the late-E population of RTN/pFRG allowed the model to reproduce several experimentally observed behaviors, including quantal acceleration of abdominal late-E oscillations with progressive hypercapnia and quantal slowing of phrenic activity with progressive suppression of pre-BötC excitability (simulating the suppressing effects of opioids), as well as to predict a release of late-E oscillations by disinhibition of RTN/pFRG under normal conditions. The latter prediction has been confirmed in our experimentally studies. Our experimental and modeling studies suggest that under normal metabolic conditions the RTN/pFRG oscillator is inhibited by both the post-I population of BötC during inspiration and early-inspiratory (early-I) population of pre-BötC during inspiration. Therefore the late-E oscillations can be released by either a hypercapnia-evoked activation of chemosensitive RTN/pFRG neurons overcoming this inhibition or a hypoxia-dependent suppression of RTN/pFRG inhibition by BötC-pre-BötC circuits.

Our model proposes mechanistic explanations for the emergence of RTN/pFRG oscillations and their interaction with the brain stem respiratory network. The emerging view is that the brain stem respiratory network has rhythmogenic capabilities at multiple hierarchical levels, which allows flexible, state-dependent expression of different rhythmogenic mechanisms under different physiological and metabolic conditions and enables a wide repertoire of respiratory behaviors.
